# Applying a New Proposed Welfare Assessment Protocol to Suckler Herds from Three Different Autochthonous Breeds

**DOI:** 10.3390/ani12192689

**Published:** 2022-10-06

**Authors:** Diana Valente, George Stilwell

**Affiliations:** 1 CIVG—Vasco da Gama Research Center, EUVG—Vasco da Gama University School, 3020-210 Coimbra, Portugal; 2 Animal Behavior and Welfare Laboratory, Centre of Interdisciplinary Research in Animal Health, Faculty of Veterinary Medicine, University of Lisbon, 1300-477 Lisboa, Portugal; 3 CIISA—Animal Behaviour and Welfare Laboratory, Associate Laboratory for Animal and Veterinary Sciences (AL4AnimalS), Faculty of Veterinary Medicine, University of Lisbon, 1300-477 Lisbon, Portugal

**Keywords:** cattle, suckler herd, welfare assessment, welfare indicators, autochthonous breeds

## Abstract

**Simple Summary:**

In recent years, there has been a large increase in consumers’ demands for farm animal welfare. The Council of the European Union emphasizes that high animal welfare is an integral part of sustainable animal production. Farmers must be able to meet these requirements by assessing animal welfare on the basis of well-defined and structured protocols for each species and production system. Only then will it be possible to guarantee welfare certification throughout all the production chain, from farm to fork. This work aims to study and apply indicators already used and validated for animals with other productive aptitudes and in other regions of the world. To this end, three cattle farms in the Center of Portugal were analyzed. Each of these farms is dedicated to the production of a Portuguese autochthonous beef breed. The welfare on these farms was rated as good and excellent in most of the criteria. This evaluation will enable the farms to meet the conditions for certification and to modify some of the less positive aspects.

**Abstract:**

The welfare of farm animals has become an increasingly important issue for society, especially for consumers of animal products. Currently, there is no standardized and validated protocol in Portugal for assessing the welfare of suckler cow herds in extensive systems. This work aims to study and apply previously used indicators, based on behavior, mental status, health, body condition, and interactions with the environment. Criteria and measures were adapted from protocols for cattle in other production systems (Welfare Quality^®^, WQ) or protocols set for pasture-based cattle in New Zealand. To the WQ measures, such as body condition, absence of injuries and diseases, positive emotional state and management indicators, we added behavior when in the chute, distance to water points, positioning of ear tags, and thermal comfort. The feasibility of the protocol was assessed in herds with cows belonging to three different Portuguese autochthonous beef breeds. The welfare of the herds was considered good or excellent, with only the behavior at the chute being negatively scored in the Brava breed. The application and validation of indicators to correctly assess animal welfare all along the production chain is crucial to achieve certification, and to the identification and correction of causes of poor welfare.

## 1. Introduction

Consumers of animal products are increasingly demanding high animal welfare standards. Additionally, the European Commission and other organizations such as World Organization for Animal Health (OIE) have included animal welfare as a crucial attribute of agriculture sustainability [1]. In order to answer to these requirements, farmers and all those involved in the production of animal-derived food must establish credible animal welfare certification schemes that will reflect and endorse the best practices. 

Currently, well-validated protocols are already available for various stages of cattle production, which allows access to certification (for example, the IRTA Welfare certification: https://www.animalwelfair.com/en/ accessed on 30 August 2022). However, the absence of suckler cow evaluation schemes prevents the suitable certification of the entire production chain (from farm to fork, as suggested in the Green Deal), as is desirable. With this work, we intend to contribute to the identification of indicators that allow for the evaluation of the well-being of beef cattle kept in extensive systems, based on their behavior, mental status, health, physical condition, and relationship with both the environment and with humans. The identification and analysis of these indicators are useful for the assessment of welfare associated with the extensive production system, and may contribute to the implementation of more integrated production and establishment of management decisions that may improve the conditions in which animals are produced. In addition, these assessments will enable consumers to be informed of welfare levels throughout the beef production chain.

A reliable and truthful assessment of animal welfare should include multiple measures that reflect physical, mental, and behavioral well-being. This requires a deep knowledge of the animals’ needs, of its natural behavior, and of its biological functions [2]. The Welfare Quality^®^ protocols outlies four principles in an attempt to grasp the complexity of animal welfare: good nutrition; good housing; good health; and appropriate Behavior. Although these general standards apply to all species and production systems, the indicators that are selected to reflect the principles may have to be adapted [3]. 

In order to assess cattle welfare in intensive systems, both the beef and dairy WQ protocols [4] selected animal-based indicators whenever possible, although some resource-based indicators are also used. In this paper, we followed the same approach. Thus, animal-based measures are used to assess health, such as the presence or absence of signs of disease or integument alterations; body condition to assess prolonged hunger, but also indirectly reflects the sanitary status of the herd (presence of parasites or debilitating chronic diseases); and comfort at resting, measuring cleanliness of the animals and time to lie down, which reflect the suitability of bedding and housing structures. Behavioral needs are associated with attitudes motivated by internal stimuli that are extremely important for the welfare of the animal [5]. Therefore, evaluation of the herd as a whole is crucial for identifying both positive mental states (e.g., cohesive contacts, playing, exploring) and negative ones (e.g., frustration, stress), and this has been achieved by applying the Quality Behavior Assessment [6]. Finally, the quality of human–animal relationships is assessed, for example, by avoidance distance. On the other hand, resource- or management-based indicators are used to assess the “prolonged thirst” criterion, by looking at water availability and cleanliness, or management of pain during farm procedures. 

Several characteristics of extensive systems reduce the importance or even preclude the use of some the indicators proposed by the WQ^®^ protocols. Impracticability or unfeasibility are reasons for discharging indicators that may be replaced by others capable of adequately assessing the degree of animal welfare. Several studies have proposed new or have adapted previous indicators, so as to be used in cattle in extensive systems [3,7], although some still have to be tested in different breeds, conditions, and environments [8]. 

The objective of this study was to test an adaptation of the WQ^®^ protocol for cattle, adding indicators proposed by other studies [3,9,10,11,12,13], in three different autochthonous breeds kept all year at pasture in the center of Portugal.

## 2. Materials and Methods

### 2.1. Principles and Criteria 

The principles and criteria proposed for our study derive from those used by the Welfare Quality^®^ protocols for dairy cows and fattening cattle [4], and by protocols for extensively kept cattle developed in New Zealand [3,9,12]. The four WQ Principles (good feeding, good housing, good health and appropriate behavior) were maintained, although good housing was replaced by good environment, as these animals are never housed. In addition, the 12 WQ^®^ Criteria were reduced to 8, due to the fact that some are not applicable to the extensive systems we studied. Whenever possible, animal-based indicators were preferred and selected. The reasoning behind the inclusion, change, or exclusion of the different criteria and indicators is described in Table 1. In the end, our protocol for suckler herds included four principles, eight criteria, and twenty-nine indicators (Table 1). Data regarding management and resources were obtained through a questionnaire for the producer or through consultation of records (if available) (Appendix A).

The reasoning behind the classification of those indicators not included in the Welfare Quality protocols for cattle is explained in the appendices. For those measures that are taken from the WQ^®^ protocols, the reader is advised to consult these online resources: (http://www.welfarequalitynetwork.net/media/1088/cattle_protocol_without_veal_calves.pdf (accessed on 1 August 2021 )).

The mental state or positive emotional state assessment of the herd was done using the Quality Behavior Assessment (QBA) descriptors for fattening cattle, which considers behavior, attitudes, and body language, as well as how animals interact with each other and with the environment [4]. The observations were made at two different observation points in the pasture for a total period of 20 minutes, as required by the WQ^®^ protocol. The results of the QBA were submitted to the simulator for fattening cattle, made available by the French National Research Institute for Agriculture, Food and Environment (INRAE) (https://www1.clermont.inrae.fr/wq/?simu=on (accessed on 10 November 2021).

Handling quality (principle appropriate behavior, human–animal relationship criterion) was evaluated by scoring individual behavior during handling, as well as the speed of entry and the speed of exit from the chute (Appendix B). The following scale of behaviors expressed during handling was used: 1 (very calm)—100; 2 (calm)—80; 3 (agitated)—55; 4 (very agitated)—20; 5 (escape)—0. A similar scale was applied to the evaluation of the speed of entry and exit from the chute: 1 (walk)—100; 2 (trot)—55; 3 (gallop)—0. The final score resulted from the calculation of the percentage of animals on the farm with each score as described by Lima et al. [13], Kaurivi et al. [3], and Spigarelli et al. [7]. The classification of the criterion is the mean value of the reaction to handling, entry speed, and exit speed.

The criterion origin of animals (Appendix C) was added in this protocol [18]. This indicator was classified according to the following scale, on the basis of whether the animal was born on the farm: 0 (yes)—100; 2 (no)—55. The final score resulted from the calculation of the percentage of animals born on the farm or submitted to transportation from another holding. 

**Figure A1:**
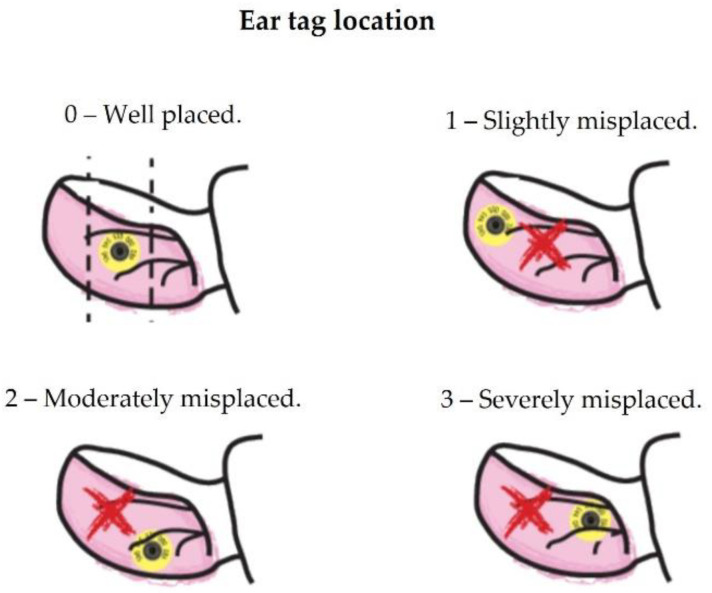
Ear tag application scoring (adapted from Richards and Gosz, n. d. [32]).

**Figure A2:**
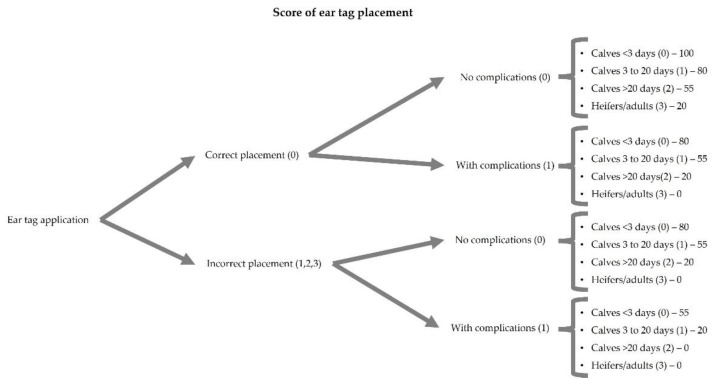
Decision tree for scoring ear tag placement.

For the principle of good health, the following WQ^®^ based criteria were used: alterations of the integument; signs of diseases; mortality in the last 12 months; pain management in painful procedures. The indicator “application of the ear tag,” related to the latter criterion, was added and classified according to the algorithm illustrated in Figure A1 and Figure A2 (Appendix D). Animals were assessed when in the chute, and the percentage of animals with well-applied or ill-applied ear tags (e.g., placement of ear tags on ridges) or with visible complications (e.g., torn ear, abscess), was registered. The final classification for each criterion resulted from the percentage of animals with each score or the percentage of animals that died at the farm in the last 12 months. The results for all WQ^®^ criterions were submitted to the INRAE simulator (see above) for dairy cows.

**Figure A3:**
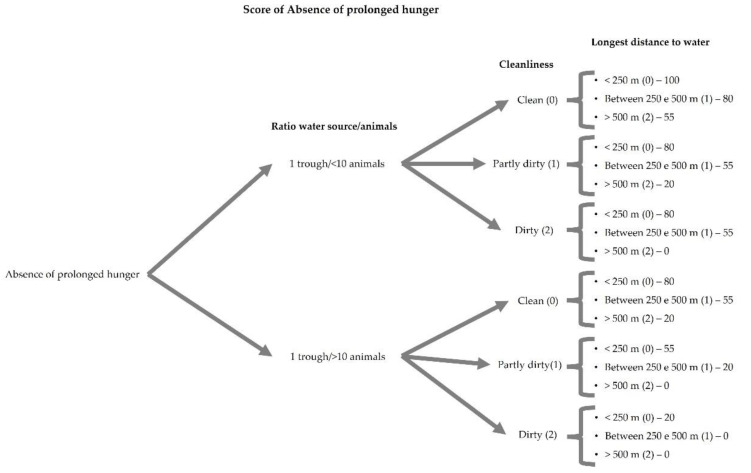
Decision tree for water availability. Having a lagoon or river available was considered as having 1 trough for <10 animals.

The principle of good feeding assesses the absence of prolonged hunger and the absence of prolonged thirst. The absence of hunger was evaluated using BCS, as proposed by the Welfare Quality^®^ protocol for fattening cattle. The evaluation of the absence of prolonged thirst was based on the following: availability of water, cleanliness of the water points, and the distance that animals had to travel to access drinkable water (Appendix E). Herd classification for this Criterion was based on the decision tree presented in Figure A3 (Appendix E).

Finally, the good environment principle was evaluated using only the thermal comfort criterion, classified through the measure “availability of sufficient shade” (Appendix F). This concept was defined as the availability of 3.2m^2^ of shade per animal. The lowest value suggested in the bibliography for grazing animals was used, because these herds were not comprised of only adult animals but also a large number of young animals that require less shade compared to adults [20].

### 2.2. Farms, Breeds and Animals

For the evaluation of the feasibility of the proposed protocol, three distinct farms were studied, with a total of 301 animals, between September 1 and October 30, 2021. These farms are dedicated to the production of cattle of three Portuguese indigenous breeds—Brava de Lide (Farm B; *n* = 161 animals), Cachena (Farm C; *n* = 123 animals) and Jarmelista (farm J; *n* = 17 animals), permanently on pasture in the Baixo Mondego region. 

The Brava de Lide breed is produced with the aim of obtaining a behavioral pattern that includes characteristics such as bravery, nobility, and “bull-fighting,” among others, not being exactly a beef breed. In contrast, the Cachena breed is one of the main cattle breeds raised in the North of Portugal, having its origin in the Peneda Gerês National Park. The typical farm regime of this breed is a mountain extensive regime, and its productive aptitude is triple (meat, milk, and work), although, nowadays, it tends to be produced mainly as a beef breed [23]. As for the Jarmelista breed, it has its origin in the region of Jarmelo, municipality of Guarda, and is reared in areas characterized by high altitude pastures, in an extensive exploitation system where small holdings predominate [24]. Both the Cachena and the Jarmelista breeds are considered endangered.

All of the indicators and criteria were analyzed and scored from 0 to 100, by which values <20 are considered as “insufficient;” values between 20 and 55 are “acceptable;” values between 55 and 80 are classified as “enhanced;” and scores above or equal to 80 are categorized as “excellent” [4,10]. Finally, the farm score is the simple mean of the four principles scores (adequate behavior, good health, good feeding, and good environment).

### 2.3. Ethic Committee Approval

Ethical review and approval were waived for this study due to the fact that the study included only observation of animals’ behavior and physical examination at a distance, both in the animals’ normal environment (pasture) or when handled in the chute for farm routine procedures. The Ethic Committee for Research and Teaching (Comissão de Ética para a Investigação e Ensino–CEIE) of the Faculty of Veterinary Medicine–-University of Lisbon declared that this type of procedure does not raise any ethical concern and that a special permission by the Committee was deemed unnecessary. The document signed by the Committee President is available for consultation. 

## 3. Results

The implementation of the proposed protocol began by evaluating the adequate behavior principle, by assessing the criteria positive emotional state and reaction to animal handling. The assessment of the positive emotional state was achieved by applying the Quality Behavior Assessment (QBA) as proposed in the WQ protocol. It was found that the three farms reached a status of excellent in this criterion, which gives us an indication of a very positive emotional state in these herds (Table 2).

As for human–animal relationships, it was found that farm B reached the enhanced level for reaction during restraint and acceptable for speed in entry and exit from the chute, with the other farms reaching the excellent score in all indicators. To summarize, the final score for the criteria handling of animals (simple mean of the two measures), was excellent for farms C and J, while farm B was classified as Enhanced (Table 3).

Finally, in the adequate behavior principle, the congregated classification of positive emotional state and animal handling, was determined (Table 4). Two farms (C and J) reached the excellent level, while farm B was considered enhanced.

As for the absence of disease and absence of injuries criteria, both farms B and C achieved the excellent score while farm J was classified as enhanced (Table 5).

Included in the good health principle, the absence of pain criterion induced by management procedures included disbudding/dehorning and a novel indicator—ear tagging. All farms were scored excellent, as no farm disbudded or dehorned their animals, and ear tags were mostly applied correctly at a very young age and no major complications were observed (Table 6).

The new indicator, animal source, was scored as excellent in two farms, which had only a few animals brought from elsewhere, but farm J was scored as enhanced, as 53% of the animals had been transported from another farm (Table 7).

Finally, the classification for the good health principle was calculated as the simple mean of the different criteria scores. All farms were considered excellent (Table 8).

In the good feeding principle, two criteria were evaluated: absence of prolonged hunger and absence of prolonged thirst. For the first, the Welfare Quality^®^ Body Condition Score was used, and all farms were classified as excellent (Table 9).

Regarding the absence of prolonged thirst, the assessment was based on the availability of water, the cleanliness of the water points, and the distance animals had to travel to get to the closest water point. Regarding the availability and cleanliness of water, this was considered maximum on farms B and C, although it was not possible to calculate the ratio of water points per number of animals due to the fact that the water source was a river, confining large areas of the pasture where the animals are kept. This is a permanent watercourse (available all year round), which always features clean water. As for farm J, only one water point was available for 17 animals, and the water was partially dirty (drinkers dirty but water fresh and clean). The distance to water points was relatively short for farms B and J, but was further away for the animals in farm C. The final classification was different for the three farms: one excellent, one enhanced, and one only acceptable (Table 10).

For this principle, the final score was calculated by the simple mean of the values of the two criteria. Two farms were scored excellent, but farm J was scored enhanced (Table 11).

Finally, the good environment principle and the thermal comfort criterion were assessed using only one indicator—shade or shelter provision. For this measure, all farms were considered excellent, as large areas of shade were present in all pastures (Table 12).

**Figure 1:**
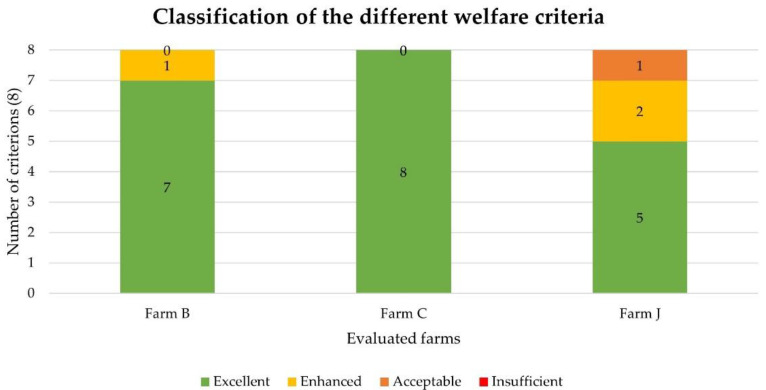
Number of criteria in each score level per farm.

In summary, the farm with the best level of welfare according to our protocol was farm C, in which all criteria achieved an excellent score. As for farm B, one criterion obtained the enhanced classification. Finally, farm J showed lower-quality results, with one criterion being classified as acceptable and two as enhanced (Figure 1).

In a global way, considering that the four principles have the same weight in the final evaluation, all farms were graded excellent, although with farm C being better scored, followed by farm B, and then farm J (Table 13).

## 4. Discussion

The application of the proposed protocol allows for the categorization of suckler-herd farms into four welfare levels: excellent, enhanced, acceptable, and insufficient [4]. These levels result from scoring the four principles (appropriate behavior, good health, good feeding, and good environment) following the scoring of different criteria, as suggested by Welfare Quality ^®^ Assessment protocol for cattle [4]. This allows farmers to recognize what needs to be amended in each farm, but also, on a more global level, it may provide support for groups of farms to achieve welfare certification for their products [25].

In the area of appropriate behavior, assessed on the basis of two criteria—positive emotional state and animal handling—all farms reached the excellent level, although farm B, in the animal handling criterion, only reached the enhanced category. This gives us an indication that there is some space for improvement. However, two facts should be considered: firstly, the same stockpersons work with the three herds, so no human-related effects are expected to influence these results; secondly, farm B is composed of animals of the autochthonous breed Brava de Lide. This breed was selected for centuries to promote its agonistic behavior and its aptitude for fight [26]. Thus, aggressive behavior during handling and high speed when entering and exiting the chute is expected for this breed. This shows that some indicators of human–animal relationships are not entirely dependent on animal management quality, but also on animals’ genetically-determined temperament.

On the other hand, although the average classification of the criterion of animal handling was excellent for both farms C and J, there was a slight difference in scores associated with reaction during handling. It would be interesting to evaluate the mean age of the animals in each herd, which would be directly related to the number of times the animals were subjected to procedures in the chute. This is one of the few factors that seems to vary between these two herds, but a more detailed analysis would have to be conducted in a future study.

Regarding the good health principle, all farms were graded as excellent, although farm J only achieved the enhanced level in the “absence of injuries and diseases” and “animals’ origin” criteria. This is a small farm with only 17 adult animals, so an injury detected in a single animal immediately translates into significant prevalence. This was exactly the case—one cow with a horn lesion represented 6% of the herd, and one occurrence of dystocia equated to 12% of calvings in the last 12 months. As for the criterion associated with the animals’ source, it was found that only 47% of the animals were born at the farm. This is due to the fact that this is a recent holding, only 3 years old, which began with the acquisition of 8 heifers and 1 steer. Furthermore, the Jarmelista breed is considered endangered, and therefore, not many purebred animals are available to be bought. Anyhow, because animals coming from other farms are necessarily subject to transport, which is a significant source of stress, it was considered to be an important indicator that should not be disregarded. According to Damtew et al. [18], the duration of the transport should also be assessed and scored. Thus, it would be interesting, in future studies, to include in the criterion score whether the transport was short- or long-term.

In the absence of injuries and diseases criterion, it would be preferable to classify claudication as mild, moderate, or severe, as is advocated by the WQ^®^ protocol for dairy cows. In our protocol, we decided to use the WQ^®^ gradation for fattening cattle, which only distinguishes non-lame from lame animals. By using the three levels’ approach, it would be easier to grade the importance of this disorder on each farm and to identify its causes, so as to recommend mitigating measures. Likewise, it would also be useful to understand the causes of diarrhea, dystocia, and horn lesions. Diarrhea may be a sign of disease or of nutritional errors (e.g., reduced fiber or excess starch) but may also simply result from a natural diet [12]. In the case of abundant, spring-fresh pasture, more fluid stool is to be expected, although no welfare issue is really present. Therefore, knowledge as to the cause of liquid feces would be advantageous for the selection of preventive measures. Regarding the occurrence of a high level of dystocia, a thorough investigation on reproduction management (e.g., heifers’ size at first breeding; sire breed; selection for calving ease) should be initiated [27]. 

Regarding mortality, another measure considered in the WQ criterion of absence of injuries and diseases, it would be interesting to understand whether on-farm deaths originate from disease or from accidents. It may happen that animals are kept in hazardous areas (e.g., cliffs, ditches, streams) or are not watched adequately, predisposing them to deaths or serious injuries that require euthanasia or emergency slaughter on the farm [14]. Again, by identifying the problem through the protocol, actions to reduce it may be implemented, contributing to overall animal welfare.

In the criterion of absence of pain induced by management procedures, all farms under study received the maximum classification, for both the WQ^®^ proposed procedure (disbudding/dehorning) as well as for the new suggested one—application of ear tags. Because dehorning is not performed on these farms, maximum classification was achieved for all farms. In contrast, application of ear tags is mandatory by law, so not applying them is not an alternative. However, applying the tags at an older age or misplacing it (ear tags on ridges or over ear cartilages), may cause unnecessary pain and complications (infection, ear tear, etc.). Ear tagging quality is a scientifically validated indicator of good husbandry practices leading to better welfare [28]. In all three farms, both the age and the placement were generally adequate. In future studies, it would be interesting to consider the impact on welfare of another form of animal identification that is used in on one of the farms under study—hot branding. This is a form of identification commonly used in Portugal on Brava de Lide cattle, and also on horses. Its application is typically carried out in young animals on a visible body region, which facilitates their identification at a distance. Alternatively, cold branding could be used, if scientific studies demonstrates a lesser impact on animal welfare.

As for the good feeding principle, only two farms reached an excellent score (B and C) while farm J was graded as enhanced. In the absence of prolonged hunger criterion, all farms reached the top classification, with no very thin animals being present. Regarding absence of prolonged thirst, farm C did not receive the maximum classification because the animals had to travel between 250 and 500 m in order to have access to water, considering the farthest point of the pasture where they are located [3]. As for farm J, it only reached the acceptable level, as the animals have access to only one water point (one water point for 17 animals), which was partially dirty. In both of these cases, there are easy opportunities for improvement, namely the installation of extra water points and close monitoring and management of all water troughs. 

Finally, for the good environment principle, all farms reached the excellent level. This was due to the fact that they all had sufficient shade for the animals in the herd. Although heat stress may have a very strong impact on health, productivity, and well-being of cattle, and this can be mitigated by providing enough shade or shelter, it would be interesting to evaluate other environmental conditions as well. Thus, in the case of extensive production systems, it is suggested that, in future studies, the number of days in which the animals are subject to extreme environmental conditions is considered. This indicator should signal not only high temperatures, but also low temperatures, as well as periods of intense and lasting rainfall, the negative impact of which can be mitigated by implementing adequate drainage systems that minimize mud formation, or by shelter building [29,30].

This study was applied to only three cattle farms in the Baixo Mondego region, which did not allow for full validation of some measures proposed, but provided a significant contribution to the much-needed inclusion of welfare assessments of suckler herds for the farm to fork certification. There is a need for wider application in other regions, with other breeds and including more indicators, as suggested above. Only after the completion of this process, with the validation of all indicators and the evaluation of their feasibility and repeatability in different settings, can a final protocol be proposed. In this process, it would be interesting to include not only the participation of farmers and auditors, but also of consumers.

## 5. Conclusions

This study aimed to identify criteria for assessing the well-being of beef cattle raised in extensive systems, through their behavior, mental status, health, physical condition, and environment to which they are subjected. In order to accomplish this, published and well-established protocols, in addition to the experience of the authors in cattle behavior and welfare assessment, were used. The final protocol was applied to three distinct farms, from which all data were collected and analyzed.

The proposed protocol consists of indicators applied previously, which were applied for cattle produced in other systems and for other purposes. For example, mental status, quality of animal handling, absence of injuries and diseases, absence of hunger and of prolonged thirst, and thermal comfort were some of the selected indicators. Nevertheless, new indicators were introduced and evaluated, such as the origin of the animals, provision of shade, and the correct application of ear tags [28], due to the fact that there is strong scientific evidence associating these factors with poor welfare. In future studies, it is proposed that a correlation between the data collected in animal handling and the mean age of the animals be established, and that the origin of the animals be assessed, taking into account whether they were subjected to short- or long-term transport. In addition, the origin of some injuries and diseases, as well as mortality causes, should be identified. For the criterion associated with painful procedures, it is proposed that animal branding be included in the assessment, as it is still used on some beef breeds, and because it causes acute and probably chronic pain leading to a permanent lesion (scar tissue). Finally, in the field of environment conditions, it is proposed to consider the numbers of days on which animals are subjected to extreme environmental conditions. 

The correct identification and interpretation of these indicators and criteria when applying this protocol to suckler herds kept in extensive systems will contribute to the implementation of more integrated beef cattle production (breeding at pasture, fattening in intensive farms, transport, and slaughter) and to the improvement of management and conditions under which animals are produced. In addition, it will meet the current requirements of consumers of animal products, who demand that the highest standards of animal welfare are attained and certified, from farm to fork.

## Figures and Tables

**Table 1:** Criteria and indicators used in the current study, based on the Welfare Quality^®^ assessment protocol for cattle [4], as well as the reason for new inclusions or exclusions.

Criteria and Indicators of Animal Welfare			
**Principle: Adequate Behavior**			
**Criteria**	**Indicators**	**Description**	**Reason for Inclusion or Exclusion**
Expression of social behaviors	Agonistic behaviors	Behaviors associated with hierarchy establishment, including head butting and fighting. High frequency may be associated with high animal density [2]. Included in both WQ protocols for cattle.	Excluded because it demands prolonged time of observation, and because extensive systems * rarely involve high animal density leading to agonistic behavior [3].
Expression of social behaviors	Cohesive behaviors	Social behaviors, indicative of good animal relationships. Included in the WQ protocol for fattening cattle [13].	Excluded because it requires prolonged observation time over a very widespread area and because there is a possibility that some factors could have a negative impact on the conclusions obtained. [13,14].
Positive emotional state	Quality Behavior Assessment (QBA)	Assesses opportunity for animals to experience positive emotions, positive affective involvement, quality of life and happiness. Included in both WQ protocols for cattle [4].	Qualitative and Behavioral Assessment included as proposed in the WQ protocol for dairy cows [4].
Good human–animal relationship	Flight distance	Measures the mean distance at which the animal retreats from human approach. Included in both WQ protocols for cattle [4].	Excluded because, in extensive systems, handling is rare and animals cannot be approached. Operator’s safety is also an issue [3].
Good human–animal relationship	Handling - behavior in chute	Reaction during handling and speed getting in and out of chute [7,13,14,15,16,17]. It assesses behavior, human handling and animals’ mental state.	New. Not assessed in the WQ^®^ protocols.
**Principle: Good Health**			
**Criteria**	**Indicators**	**Description**	**Reason for inclusion or exclusion**
Absence of diseases	% of animals with the condition during the last 12 months	Mortality, included in both WQ protocols for cattle. Dystocia, downer cow, somatic cell counts. Included in WQ protocol for dairy cows [4].	Evaluation based on farm records and questionnaires applied to the farmer as in WQ protocols. All included except for somatic cell count and downer cow [4].
Absence of diseases	Signs of different diseases	Diarrhea. Nasal, ocular, vulvar discharges. Coughing [4].	Assessment as suggested in both WQ protocols [4].
	Integument alterations	Lesions and hairless patches [4].	Assessment as suggested in the WQ protocols [4].
Absence of pain induced by management procedures	Dehorning and disbudding. Tail docking. Castration.	Method used. Use of anesthesia and analgesia. Questionnaire [4].	Dehorning/disbudding as suggested in both WQ protocols. Exclusion of castration and tail docking because these are not performed in suckler herds.
	Application of ear tags	Age when applied (questionnaire). Ear location where tag is applied by animal examination when in chute.	New. Not assessed in WQ protocol but suggested by Kaurivi et al. [3,17].
Animal source		Farm of origin and the need for transport [18,19]. Records or questionnaire.	New. Included as suggested by Damtew et al. [18].
**Principle: Good Nutrition**			
**Criteria**	**Indicators**	**Description**	**Reason for exclusion**
Absence of prolonged hunger	Body Condition Score	Evaluation of the body condition of the adult animals in the herd. Included in both WQ protocols for cattle [4].	Assessed as suggested by WQ protocols [4].
Absence of prolonged thirst	Quality of water source	Assessment of the number and size of available water points, water cleanliness, and distance animals have to travel to access the water [3,4].	Resource-based indicator. Cleanliness and size as in the WQ protocols [4]. Accessibility as suggested by Kaurivi et al. [3].
**Principle: Good Environment**			
**Criteria**	**Indicators**	**Description**	**Reason for inclusion or exclusion**
Thermal comfort	Provision of sufficient shade	Shade availability (trees, hedges, barriers, shelters, etc.) in area sufficient for all animals [12,20,21].	Resource-based indicator. Presence or absence as suggested by Kaurivi et al. [11] and Edwards-Callaway [20].
Comfort around resting	Body cleanliness	Area with solid or liquid dirt. Included in both WQ protocols for cattle [4].	Excluded because of low prevalence and because it may not reflect bedding quality/wet grass (Kaurivi et al.) [3].
	Time to lie down in seconds	Time to lie down. Included in both WQ protocols for cattle [4].	Excluded as not applicable to animals lying in an open field.
Ease of movement	Access to pasture	Days per year and hours per day with access to loafing area or pasture [4].	Days per year included as suggested by [4]. Hours per day not applicable as animals are in pasture all day.

* Extensive systems: production using grazing as part of the production process and whose stocking density does not exceed 1.4 Livestock Unit/ha (LU/ha), this value may be extended up to 2.8 LU/ha, provided that two thirds of the grazing livestock’s food requirements are met. Livestock Unit is the standard unit of equivalence used to compare and aggregate numbers of animals of different species or categories, taking into consideration the animal species, age, live weight, and production, in relation to food requirements and the production of livestock effluents [22].

**Table 2:** Quality Behavior Assessment (QBA) on the three farms belonging to the study (values are in mm in a 125mm visual analogue scale).

Application of Quality Behavior Assessment (QBA)			
QBA	Farm B	Farm C	Farm J
**Active**	73	97	57
**Indifferent**	74	62	110
**Nervous**	0	0	0
**Relaxed**	112	120	120
**Frustrated**	0	0	0
**Agitated**	9	0	0
**Uneasy**	0	0	0
**Friendly**	34	111	96
**Calm**	103	110	109
**Bored**	0	0	0
**Sociable**	35	109	107
**Content**	102	117	107
**Positively occupied**	108	119	108
**Happy**	111	117	118
**Distressed**	0	0	0
**Inquisitive**	48	97	76
**Distressed**	0	0	0
**Lively**	97	99	92
**Irritable**	5	0	0
**Final Score**	**86**	**100**	**91**

**Table 3:** Scoring of the three farms in reaction to handling and in speed at entry and exit from chute.

Human–Animal Relationship Assessment			
Indicators	Farm B	Farm C	Farm J
**Reaction During Handling (% of Animals in Herd)**			
1 (very calm)	19%	84%	59%
2 (calm)	56%	11%	29%
3 (agitated)	19%	5%	6%
4 (very agitated)	4%	0%	6%
5 (escape)	2%	0%	0%
Indicators score (mean)	2.15	1.21	1.59
Criteria Score	76	96	88
**Speed Entering and Exiting the Chute (% of Animals in Herd)**			
1 (walk)	8%	85%	88%
2 (trot)	51%	11%	6%
3 (gallop)	41%	4%	6%
Indicators score (mean)	2.329	1.195	1.176
Criteria Score	37	91	92
**Final score for the two indicators**	**57**	**91**	**90**

**Table 4:** Final score for the adequate behavior principle.

Adequate Behavior Principle			
Indicators	Farm B	Farm C	Farm J
Positive Emotional State	86	100	91
Animal handling	57	91	90
**Final Score for the Principle**	**72**	**96**	**91**

**Table 5:** Results for the indicators in absence of disease and absence of injuries in the three study farms.

Absence of Disease and Injuries (Percentage of Animals on the Farm)			
Indicators	Farm B	Farm C	Farm J
Lameness	1%	2%	0%
Hairless patches/lesions	2%	1%	0%
Horn lesions	4%	2%	6%
Coughing	0%	0%	0%
Nasal Discharge	0%	0%	0%
Ocular Discharge	0%	0%	0%
Hampered respiration	0%	0%	0%
Diarrhea	1%	1%	0%
Vulvar discharge	0%	0%	0%
Dystocia	0%	0%	14%
Mortality	3%	2%	0%
**Final Score (mean)**	**88**	**99**	**72**

**Table 6:** Results regarding disbudding/dehorning and ear-tagging.

Disbudding/Dehorning and Ear-Tagging			
Indicators	Farm B	Farm C	Farm J
**Disbudding/Dehorning**			
Method	0–100%	0–100%	0–100%
Anesthesia	Not applicable	Not applicable	Not applicable
Analgesia	Not applicable	Not applicable	Not applicable
**Ear Tagging**			
Tag placement	0–98% 2–2%	0–99% 2–1%	0100%
Complications	0–100%	0–100%	0–100%
Age at ear tagging	0–100%	0–100%	0–100%
**Final score (mean)**	**100**	**100**	**100**

**Table 7:** Results of the three farms regarding the origin of their animals.

Animal Source				
Indicators		Farm B	Farm C	Farm J
**Born at the farm**	Yes (0)	90%	85%	47%
	No (2)	10%	15%	53%
**Final score (mean)**		**96**	**93**	**79**

**Table 8:** Final score for the good health principle.

Principle Good Health			
Criteria	Farm B	Farm C	Farm J
Absence of disease and lesions	88	99	72
Painful procedures	100	100	100
Animal source	96	93	76
**Final score (criteria simple mean)**	**95**	**97**	**83**

**Table 9:** Body Condition Score in the three farms using the WQ^®^ evaluation system.

Absence of Prolonged Hunger			
Indicator	Farm B	Farm C	Farm J
Body Condition Score (% of very thin animals—2)	0%	0%	0%
**Final score**	**100**	**100**	**100**

**Table 10:** Results from the assessment of water availability and cleanliness.

Absence of Prolonged Thirst			
Indicators	Farm B	Farm C	Farm J
Nº water points	1 drinker/<10 animals (River)	1 drinker/<10 animals (River)	1 drinker/>10 animals
Cleanliness	Clean	Clean	1–Partially dirty
Distance to water	0–<250 m	1–Between 250 m e 500 m	0–<250
**Final score (mean)**	**100**	**80**	**55**

**Table 11:** Classification of the good feeding principle.

Good Feeding Principle			
Criteria	Farm B	Farm C	Farm J
Absence of prolonged hunger	100	100	100
Absence of prolonged thirst	100	80	55
**Final score (mean)**	**100**	**90**	**78**

**Table 12:** Assessment of thermal comfort by way of shade provision.

Thermal Comfort			
Indicator	Farm B	Farm C	Farm J
Provision of shade	0—Enough shade	0—Enough shade	0—Enough shade
**Final Score (mean)**	**100**	**100**	**100**

**Table 13:** Final classification of farms accordingly to the four principles.

Final Classification			
Principle	Farm B	Farm C	Farm J
Appropriate Behavior	72	96	91
Good Health	95	97	83
Good Feeding	100	90	78
Good Environment	100	100	100
**Final Score**	**92**	**96**	**88**

## Data Availability

The data presented in this study are available on request from the corresponding author.

## Appendix A. Questionnaire for the Farmers (Translated from Portuguese by the Authors)

This questionnaire was developed within the research work entitled "Applying a new proposed welfare assessment protocol to suckler herds from three different autochthonous breeds." The objective of this study was to test an adaptation of the WQ^®^ protocol for cattle, adding indicators proposed by other studies. The questions are intended to be answered by cattle breeders, and the results obtained will be used for scientific purposes only. The questionnaire is anonymous, so please do not use your personal identification in any of the questions. Thank you very much for your cooperation!

Farm________________________________________________________________________

Number of animals __________ Breed _________________

1. **Animal source**

1.1 How many animals in your herd were born on your own farm?

________ animals

1.2 How many animals in your herd were born on another farm?

________ animals

2. **Handling procedures—dehorning and disbudding:**

2.1 Do you perform dehorning/disbudding on your farm?

◯ Yes  ◯ No

If you answered YES:

2.2 Method used:

2.2.1 Is disbudding of calves performed using thermocautery?

◯ Yes  ◯ No

2.2.2 Is disbudding of calves performed using caustic paste?

◯ Yes  ◯ No

2.2.3 Is dehorning performed on adult animals?

◯ Yes  ◯ No

2.2.4 Do you use anesthesia when performing dehorning/disbudding?

◯ Yes  ◯ No

2.3 Do you use post-surgery analgesics when performing dehorning/disbudding?

◯ Yes  ◯ No

3. **Handling procedures—application of ear tags:**

3.1 At what age are you tagging your animals?

◯ Calves up to 3 days.

◯ Calves 3 to 20 days old.

◯ Calves older than 20 days and up to 6 months old.

◯ Animals older than 6 months.

4. **Absence of diseases**

4.1 How many instances of dystocia (percentage of difficult calvings) occurred on your farm during the past 12 months?

______________ dystocia

4.2 Mortality

4.2.1 How many animals died (disease or accident) on your farm during the last 12 months?

______________ animals

4.2.2 How many animals were euthanized on your farm during the last 12 months?

______________ animals

4.2.3 How many animals were subject of emergency slaughter during the last 12 months?

______________ animals

## Appendix B. Behavior during Containment and Speed of Entry and Exit in the Chute

The evaluator records the behavior of each animal during containment for routine procedures (e.g., vaccination) and measures the speed of entry and exit from the chute, using the following scoring system:

Behavior during handling [7,13,15,16]:

1. (Very calm)—the animal remains calm, without moving the body or limbs, during the performance of the procedure;

2. (Calm)—the animal moves the head and body gently while procedures are being performed;

3. (Agitated)—the animal moves the head, body, and limbs during procedures frequently and effectively;

4. (Very agitated)—the animal moves very frequently, rapidly, and forcibly, shaking the body and lifting the limbs during the performance of procedures;

5. (Escape)—the animal struggles to escape from the chute, lifting both front limbs and behaving aggressively during the procedures.

Speed of entry and exit from the chute [7,13, 15,16]:

1 (walk)—four-beat gait characterized by the progression of the limbs alternately, in which each hits the ground independently.

2 (trot)—two-beat diagonal gait, in which the limbs move in pairs diagonally, but not exactly simultaneously.

3 (gallop)—three-beat march, in which the forelimbs hit the ground quickly, one after the other, followed by the pelvic limbs.

Herd level classification is the percentage of animals with each score.

## Appendix C. Animal Source

The data collected in relation to the origin of the animals should be based on existing records [18]. Thus, records should be evaluated to determine how many animals were born outside the farm. Individual classification is performed according to the following criteria, on the basis of whether the animal was born on the farm:

0—yes.

2—no (transported to the farm).

Herd classification is the percentage of animals born outside the farm.

## Appendix D. Absence of Pain Induced by Management Procedures

Ear tagging

The data collected in relation to the application of ear tags is based on a questionnaire applied to the farmer and on the direct observation of the animals. The farmer will indicate the age at which ear tagging usually occurs. Animal examination during the time it is in the chute will register tag placement and presence of complications, such as a torn ear or infection. Scoring is performed through the following system [3,31,32]:

(a) Ear tag location:

(b) Presence of complications due to ear tagging:

0—no complication—ear hole is round, small, clean, and dry.

2—complications evident—ear torn, presence of exudate, presence of more than one hole, pus, etc.

(c) Age at ear tagging:

0—calves up to 3 days.

1—calves 3 to 20 days old.

2—calves older than 20 days and up to 6 months old.

3—animals older than 6 months.

Taking these three parameters into account, the herd scoring results from the following algorithm:

## Appendix E. Absence of Prolonged Thirst

Distance to water points:

The distance that animals have to travel to access the water points at the farthest point of the area where they are kept is measured. The classification is performed according to the following criteria (3,12):

0—animals have to walk up to 250 m.

1—animals have to walk between 250 and 500 m.

2—animals have to walk >500 m (3, 12).

Herd level classification is obtained through the following decision tree:

## Appendix F. Thermal Comfort

The evaluation of thermal comfort was performed through a resource based measure—calculation of total area with shade (trees, barriers, hedges, barns, or shelters). Classification should be carried out according to the following criteria [12]:

0—shade is sufficient (≥3.2 m^2^/animal).

2—shade is not sufficient (<3.2 m^2^/animal).
